# Reproducibility of Targeted Lipidome Analyses (Lipidyzer) in Plasma and Erythrocytes over a 6-Week Period

**DOI:** 10.3390/metabo11010026

**Published:** 2020-12-31

**Authors:** Marieke Loef, Johannes H. von Hegedus, Mohan Ghorasaini, Féline P. B. Kroon, Martin Giera, Andreea Ioan-Facsinay, Margreet Kloppenburg

**Affiliations:** 1 Department of Rheumatology, Leiden University Medical Center, Albinusdreef 2, 2300 RC Leiden, The Netherlands; j.h.von_hegedus@lumc.nl (J.H.v.H.); f.p.b.kroon@lumc.nl (F.P.B.K.); aif@genmab.com (A.I.-F.); g.kloppenburg@lumc.nl (M.K.); 2 Center for Proteomics and Metabolomics, Leiden University Medical Center, Albinusdreef 2, 2300 RC Leiden, The Netherlands; m.ghorasaini@lumc.nl (M.G.); m.a.giera@lumc.nl (M.G.); 3 Department of Clinical Epidemiology, Leiden University Medical Center, Albinusdreef 2, 2300 RC Leiden, The Netherlands

**Keywords:** lipids, metabolomics, reproducibility, plasma, erythrocytes

## Abstract

It is essential to measure lipid biomarkers with a high reproducibility to prevent biased results. We compared the lipid composition and inter-day reproducibility of lipid measurements in plasma and erythrocytes. Samples from 42 individuals (77% women, mean age 65 years, mean body mass index (BMI) 27 kg/m^2^), obtained non-fasted at baseline and after 6 weeks, were used for quantification of up to 1000 lipid species across 13 lipid classes with the Lipidyzer platform. Intraclass correlation coefficients (ICCs) were calculated to investigate the variability of lipid concentrations between timepoints. The ICC distribution of lipids in plasma and erythrocytes were compared using Wilcoxon tests. After data processing, the analyses included 630 lipids in plasma and 286 in erythrocytes. From these, 230 lipids overlapped between sample types. In plasma, 78% of lipid measurements were reproduced well to excellently, compared to 37% in erythrocytes. The ICC score distribution in plasma (median ICC 0.69) was significantly better than in erythrocytes (median ICC 0.51) (*p*-value < 0.001). At the class level, reproducibility in plasma was superior for triacylglycerols and cholesteryl esters while ceramides, diacylglycerols, (lyso)phosphatidylethanolamines, and sphingomyelins showed better reproducibility in erythrocytes. Although in plasma overall reproducibility was superior, differences at individual and class levels may favor the use of erythrocytes.

## 1. Introduction

Lipidomics analysis involves the identification and quantification of molecular lipid species. Lipids as part of the cellular membrane play vital roles in cellular function and signaling. Furthermore, the lipidome is a highly dynamic pool of molecules, constantly adapting to physiological and pathological conditions [1]. In turn, lipidomics analysis has become a valuable technology for understanding physiological and pathological mechanisms and the identification of candidate biomarkers. Biomarkers are indicators of normal or pathogenic biological processes that help in understanding the pathogenesis of diseases or to measure disease presence and predict disease progression. In addition, biomarkers may be used to assess or to predict the individual response to pharmacological treatments and may therefore play an important role in drug development [2,3,4]. For the correct assessment and interpretation of biomarkers, it is essential that the measurement of potential biomarkers is reliable and reproducible. It is often assumed that measurements taken on a single day are representative of the metabolic status of an individual. However, fluctuations in biomarker concentration may occur due to sampling techniques, assay variation, or biological variability [5]. Biological variability, defined as naturally occurring within-subject fluctuations in repeated measurements, may lead to bias towards the null when estimating the association between biomarkers and a disease or treatment [6]. To estimate the variability of the measurement, information regarding the stability of the metabolite levels over time is essential.

Data regarding the reproducibility of lipid metabolite measurements are scarce [7,8,9,10,11,12]. Of the available studies, most investigated technical reproducibility rather than biological variability [7,10,11], or used metabolomic platforms evaluating a relatively small number or variety of lipid metabolites [7,8,9,10,12]. The Lipidyzer platform is a commercially available targeted lipidomics platform with the potential to measure >1000 individual lipid species. While other studies thus far have mainly focused on inter-laboratory and cross-platform comparisons [13,14,15], to our knowledge, no biological reproducibility studies using plasma and erythrocytes derived from the same subjects over an extended time span (6 weeks) have yet been performed using the Lipidyzer platform, or other lipid platforms of comparable extent. In a clinical setting, serum and different types of plasma are the most commonly used sample types for metabolomic studies. However, when investigating chronic alterations of the lipidome, other sample types might be more representative. In this respect, erythrocytes display minimal cellular metabolism and have a long half-life of approximately 120 days; their membrane lipids may therefore reflect the long-term exposure of an individual, particularly relevant when studying chronic diseases or prolonged treatments. However, a detailed study comprehensively and quantitatively describing the biological reproducibility of erythrocytes has not yet been established.

Therefore, the aim of our research was twofold. Firstly, we aimed to investigate the clinically relevant inter-day reproducibility (a combination of biological and technical variability) of lipid measurements over a period of 6 weeks in plasma and erythrocytes using the Lipidyzer platform. Secondly, we compared the variety and abundance of lipid species, as well as their reproducibility between the two different sample types.

## 2. Results

### 2.1. Study Population and Samples

The study population consisted of 42 individuals. The population was predominantly composed of women (77%), with a mean (SD) age of 64.9 (8.3) years and a mean (SD) BMI of 27.1 (5.2) kg/m^2^.

**Figure 1:**
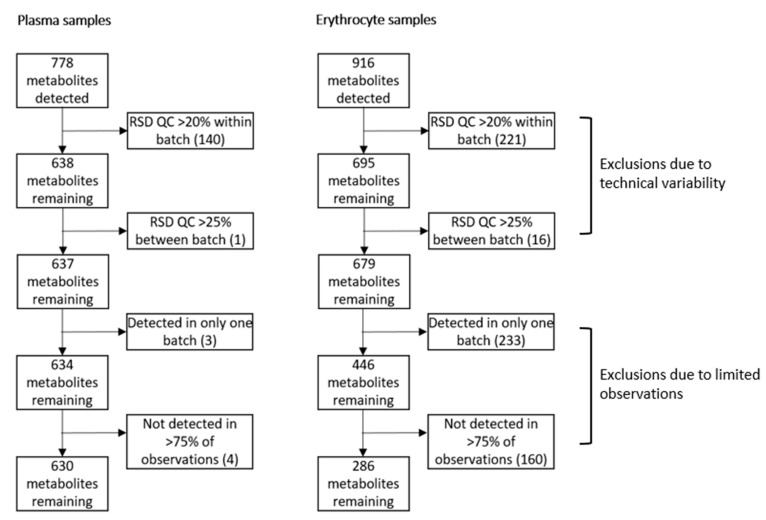
Pre-processing steps and exclusion numbers of Lipidyzer variables. Lipid metabolite variables were excluded if the relative standard deviation (RSD) of the quality control (QC) was >20% within a batch, or >25% between separate batches if a lipid was detected in one batch only or not detected in >75% of the observations.

At baseline and week 6, 31 and 33 plasma samples, and 35 and 29 erythrocyte samples, respectively, were available for analyses. The Lipidyzer platform is an integrated system with the potential to quantify over 1000 lipids across 13 lipid classes. We detected and quantified 778 distinct lipid species in plasma, while in erythrocytes, 916 lipids were quantified. Processing of the data resulted in 630 lipids in plasma, and 286 lipids in erythrocytes remaining for further analyses. Of these, 230 lipids were measured both in plasma and erythrocytes. The pre-processing steps and exclusion numbers are shown in Figure 1.

### 2.2. Lipid Composition in Plasma and Erythrocytes

**Figure 2:**
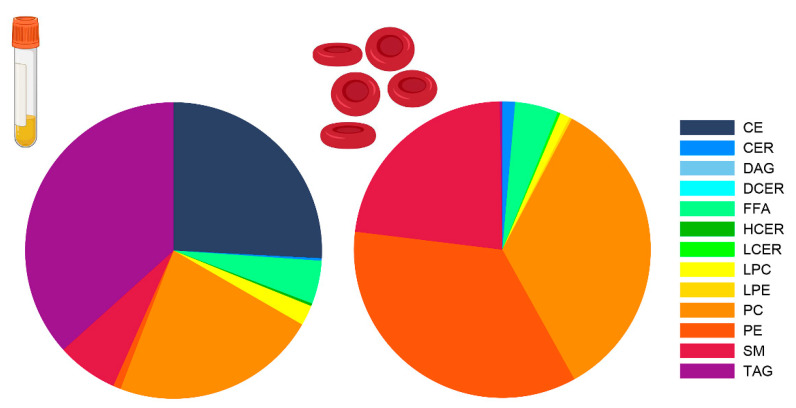
Plasma and erythrocyte lipid class composition. Lipid class composition in plasma (left) and erythrocytes (right) as a percentage of the total lipid concentration. Abbreviations: CE = cholesteryl ester, CER = ceramide, DG = diacylglycerol, DCER = dihydroceramide, FFA = free fatty acid, HCER = hexosylceramide, LCER = lactosylceramide, (L)PC = (lyso)phosphatidylcholines, (L)PE = (lyso)phosphatidylethanolamine, SM = sphingomyelin, TAG = triacylglycerol.

Major differences were observed between the sample types in the number of lipids per lipid class and the total concentration of the lipids within a class (Table 1). Triacylglycerols (TAG) were much more abundant in plasma; 482 individual TAGs were quantified with a median concentration of 1579.4 nmol/mL. In contrast, in erythrocytes, 134 TAGs were quantified with a median concentration of 6.5 nmol/mL. In addition, 24 cholesteryl esters (CE) (4571.6 nmol/mL) were quantified in plasma, while only 5 CEs (1.2 nmol/mL) were quantified in erythrocytes. Conversely, phosphatidylcholines (PC) (*n* = 31, 4013.7 nmol/mL vs. *n* = 42, 3899.2 nmol/mL), phosphatidylethanolamines (PE) (*n* = 26, 156.2 nmol/mL vs. *n* = 42, 3954.6 nmol/mL), sphingomyelins (SM) (*n* = 12, 1204.6 nmol/mL vs. *n* = 8, 2695.8 nmol/mL), and ceramides (CER) (*n* = 6, 14.1 nmol/mL vs. *n* = 7, 163.0 nmol/mL) were less abundant in plasma compared to erythrocytes. Figure 2 presents the relative composition of the lipid classes in the two sample types.

### 2.3. The Majority of Lipid Species Showed Good Reproducibility in Plasma

**Figure 3:**
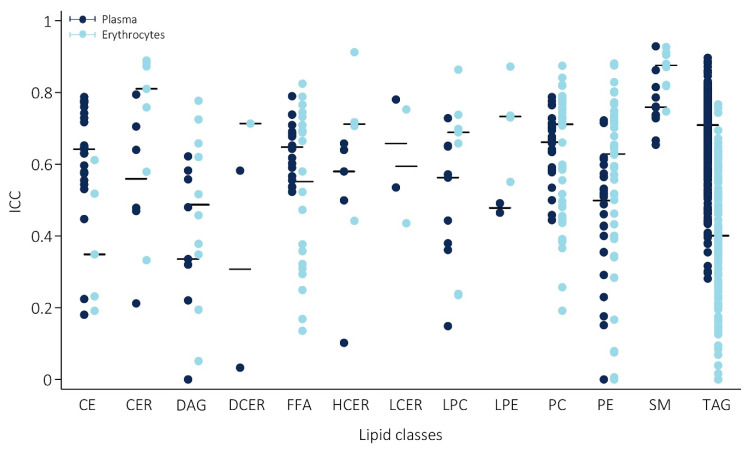
Reproducibility of the lipids over a 6-week period, stratified by lipid class. Reproducibility of individual lipids within each class in plasma (blue) and erythrocytes (red); bars represent the median intraclass correlation coefficient (ICC) of the lipid class. Abbreviations: ICC = intraclass correlation coefficient, CE = cholesteryl ester, CER = ceramide, DAG = diacylglycerol, DCER = dihydroceramide, FFA = free fatty acid, HCER = hexosylceramide, LCER = lactosylceramide, (L)PC = (lyso)phosphatidylcholines, (L)PE = (lyso)phosphatidylethanolamine, SM = sphingomyelin, TAG = triacylglycerol.

Reproducibility in plasma varied greatly between lipids, with intraclass correlation coefficients (ICCs) ranging from ICC = 1.1 × 10^−25^ for diacylglycerol (DG) at 16:0_20:4, to excellent, with the highest ICC of 0.93 for sphingomyelin (SM) at 24:0. Generally, reproducibility was good, with a median ICC of 0.69. Categorization of the individual lipids according to their degree of reproducibility showed that in plasma, only 5% of lipids were poorly reproducible and 18% were moderate, while 70% and 8% were categorized as having a good and excellent reproducibility, respectively. At the class level, the lipid classes showing best reproducibility were SMs (median ICC = 0.77) and triacylglycerols (TAGs) (median ICC = 0.72). In Figure 3, the range in ICCs of the individual lipids within each class and the median of the individual lipids for each class are given (plasma measurements are shown in blue). The ICCs and corresponding 95% CI, as well as the mean change in concentration over time of all individual lipids are provided in Table S2.

### 2.4. Comparison of Erythrocyte Lipid Reproducibility

**Figure 4:**
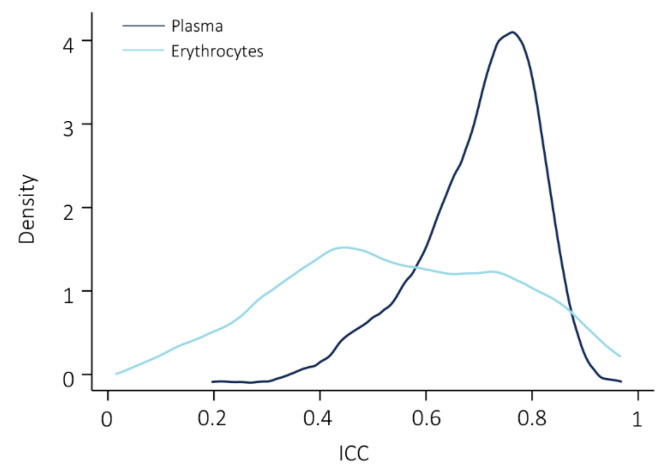
Density plot of the ICC score distribution of lipids measured in plasma and erythrocytes. The Wilcoxon signed-rank test showed a significant difference in the ICC distributions between plasma and erythrocytes, with a *p*-value < 0.001.

Similarly to plasma, reproducibility of lipid measurements in erythrocytes varied greatly, with the worst reproducibility observed for TG 54:2-FA 16:0 (ICC = 1.7 × 10^−24^) and best reproducibility for SM 26:1 (ICC = 0.93). Compared to plasma, a considerably larger amount of lipids was poorly reproducible (34%). Reproducibility was moderate in 28%, good in 28%, and excellent in 9% of lipids measured in erythrocytes. Figure 4 shows the ICC score distribution of the lipids overlapping between the sample types. Comparison of the overall reproducibility of the lipids measured in both sample types showed a significantly higher variability in erythrocytes (median ICC = 0.51) compared to plasma (median ICC = 0.70), with Wilcoxon signed-rank test *p*-value < 0.001.

However, while overall reproducibility was better in plasma, this was not observed for all lipid classes, as is evident from Figure 3. In erythrocytes, reproducibility at the class-level (median ICC of the individual lipids within a class) was notably better in CERs (median ICC = 0.81 vs. ICC = 0.56 in plasma), diacylglycerols (DAGs) (median ICC = 0.49 vs. ICC = 0.34 in plasma), (lyso)phosphatidylethanolamines ((L)PEs) (median ICC = 0.73 vs. ICC = 0.48 in plasma), PEs (median ICC = 0.63 vs. 0.50 in plasma), and SMs (median ICC = 0.88 vs. 0.76 in plasma). In contrast, plasma reproducibility was better in TAGs (median ICC = 0.71 vs. ICC = 0.40 in erythrocytes), CEs (median ICC = 0.64 vs. ICC = 0.35 in erythrocytes), and free fatty acids (FFAs) (median ICC = 0.65 vs. ICC = 0.55 in erythrocytes). Table S3 provides an additional comparison of the median ICCs of each lipid class considering only the lipids species overlapping between the two sample types (*n* = 230).

## 3. Discussion

In the current study, we aimed to investigate the clinically relevant inter-day reproducibility of lipid measurements with the Lipidyzer platform over a 6-weeks period in plasma and erythrocytes. We analyzed 630 and 286 individual lipid species in plasma and erythrocytes, respectively. In plasma, overall reproducibility of lipid measurements was good, and 78% of lipids were reproduced well to excellently. At individual lipid levels and class level, major differences in reproducibility were observed, with the best reproducibility in SMs and TAGs. Comparison of variability between plasma and erythrocytes showed a significantly better overall reproducibility in plasma. Furthermore, only 37% of lipid measurements were reproduced well to excellently in erythrocytes. However, sample type preference should be based on the individual lipids or lipid class of interest due to differences in reproducibility between sample types at individual lipid and class level.

We presented a standardized manner to pre-process the Lipidyzer data on the basis of the relative standard deviation (RSD) of the quality control samples included in each measurement run. We observed that although less lipid species were initially quantified in plasma samples, after data processing, approximately twice the number of lipids was available for further analyses in plasma compared to erythrocytes. Approximately one-third of the measured lipids overlapped between plasma and erythrocytes. As expected, the quantity of lipids, both in number and concentration, varied between sample types. In plasma, the most abundant lipids were CEs, TAGs, and PCs, which is in line with the National Institute of Standards and Technology (NIST) interlaboratory lipidomics comparison study [13]. Compared to erythrocytes, CEs and TAGs were more abundant in plasma. Conversely, PC, PE, SM, and CER were in higher number and concentrations present in erythrocytes. The higher abundance of TAG and CE in plasma likely reflects liver lipid metabolism and lipid transport through the body [16,17]. The high concentration of PC, PE, SM, and CER in erythrocytes reflects cell membrane composition, and is also in line with previous findings [18].

We showed great variability in the reproducibility between individual lipids and lipid classes, from very poor to excellent, in both sample types. Although overall the reproducibility of lipid measurements in plasma surpassed the reproducibility in erythrocytes, this was lipid- and class-specific. We observed that lipids that were most abundant within a sample type showed the best reproducibility. For example, in erythrocytes, the more abundantly present lipids such as PC, PE, SM, and CER were markedly better reproduced in erythrocytes compared to plasma, which suggests that long-term cellular membrane lipid composition may preferable be measured in erythrocytes [19].

Our results on the quantity and reproducibility of the lipid measurements imply that the preference of sample type for lipid measurements depends on the research objectives and the lipids under investigation. Research focused on lipid metabolism and transport make best use of plasma samples, while research regarding the long-term effects of interventions on cellular membrane lipid composition may benefit from the use of erythrocyte samples.

There are some notable strengths and limitations to our study. As the Lipidyzer platform uses a targeted, multiple reaction monitoring (MRM)-based technology, the here-investigated lipids are predefined by the actual analytical method. In turn, other lipid classes, for example phosphatidylserines known to be particularly present in erythrocytes, could not be targeted in our study. We used data from a randomized controlled trial, which offered the benefits of standardized hospital visits and strict monitoring, which contributed to high data quality. Although this restricted our sample size, the study population was still relatively large compared to most lipidomic studies. Our study population consisted of hand osteoarthritis patients, who were predominantly elderly women. Since hand osteoarthritis is very common in the elderly population, this likely had little influence on the observed lipid concentrations. However, the observed lipid profile may not be representative of the general population, or of younger individuals. Furthermore, data on lipid-lowering medication was not available. Besides being a reflection of variances in dietary intake and lipid metabolism, reproducibility may be influenced by variances in blood drawing, processing, and laboratory handling. In the current study, samples were obtained using a standardized operating procedure, and plasma and erythrocyte samples originated from the same initial test tube. Differences may have occurred in sample handling before and during lipid measurement, as the erythrocyte samples were measured by a different analyst (JvH) than the plasma samples (MG), approximately 1 year apart. However, the two timepoints from each sample type on which the ICCs were based had the same sample handling. Therefore, we expect any potential effects this may have on the presented results to be limited. However, the erythrocyte measurements were corrected for protein pellet, which may have introduced measurement error to some extent. Furthermore, we observed a higher RSD of the QCs in a large amount of lipid species in our erythrocyte measurements, which indicates somewhat higher technical imprecision and resulted in the exclusion of more lipid species in the erythrocyte samples. We performed the blood sampling non-fasted, with variable sampling timepoints due to differences in scheduled hospital visits, which may be viewed as a limitation. Despite this, we showed that a large proportion of the lipid metabolites had a high reproducibility. This is encouraging, as this procedure better reflects the daily practice and limits patient burden. Moreover, identification of biomarkers that do not required fasted sampling will greatly increase feasibility and implementation in large epidemiological studies.

In conclusion, we have shown that inter-day reproducibility is good to excellent for the majority of lipids in plasma. Although overall the reproducibility was better in plasma compared to erythrocytes, notable differences were observed at the individual lipid level and at the lipid class level that may favor the use of erythrocyte samples. In the evaluation of dietary intervention studies, it is essential to measure lipids with high reproducibility to prevent biased results. The presented findings may guide methodological considerations in future lipid biomarker studies.

## 4. Materials and Methods

### 4.1. Study Design and Patients

The current study included placebo-treated patients included in the Hand Osteoarthritis Prednisolone Efficacy (HOPE) study. The HOPE study was a blinded, randomized, placebo-controlled trial that investigated the effect of prednisolone treatment in patients with painful, inflammatory hand osteoarthritis. Full description of patient inclusion and procedures is described elsewhere [20]. Briefly, patients fulfilling the American College of Rheumatology criteria [21] and presenting signs of inflammation in the interphalangeal joints were included. Exclusion criteria involved chronic inflammatory rheumatic diseases, psoriasis, uncontrolled serious comorbidities, malignancy, infectious disease, and immune modulating drug use within 90 days before baseline. The HOPE study (Netherlands Trial Registry (NTR5263)) was approved by the local medical ethics committees and conducted in accordance with Good Clinical Practice guidelines and Declaration of Helsinki. All patients provided written informed consent.

### 4.2. Blood Sampling and Lipidomics Analysis

Blood samples were obtained non-fasted at baseline and after 6 weeks in EDTA tubes, following a standardized protocol. The blood samples were centrifuged for 10 min at 2200× *g* to separate plasma from the cellular fraction. Erythrocytes were isolated by Ficoll density gradient centrifugation and washed 3 times with PBS. Samples were stored at −80 °C and topped with argon until further analyses [22]. The Lipidyzer platform (Sciex) was used to quantify total lipid content in plasma (nmol/mL) and erythrocytes (nmol/mL). Lipid extraction was performed using methyl-tert-butylether as described by Matyash et al., with some modifications [23]. To 25 μL of erythrocyte sample or plasma, we added the following: 160 μL MeOH, 50μL internal standard solution (Lipidyzer internal standard kit, containing > 50 labeled internal standards for 13 lipid classes), and 550 μL methyl-tert-butylether. Samples were vortexed and left at room temperature for 30 min. Subsequently, 200 μL water was added for phase separation and the samples were centrifuged at 1,3100× *g*. The upper layer was transferred to a glass vial and lipid extraction was repeated by adding 300 μL methyl-tert-butylether, 100 μL MeOH, and 100 μL water. The organic extracts were combined and dried under a gentle stream of nitrogen. Lipidyzer running buffer consisting of 10 mM ammonium acetate in 50:50 (*v*/*v*) dichloromethane/methanol (250 μL) was added, and samples were transferred to a glass vial with insert for injection. Briefly, the Lipidyzer platform is a flow-injection-based ion-mobility triple quadrupole system consisting of a Sciex 5500 QTrap equipped with SelexIon technology coupled to a Shimadzu Nexera series UHPLC system used for injection and delivering running buffer at 7 µL/min. A total of 50 μL of the resuspended samples were injected using 2 dedicated methods. Method #1 operated with active DMS separation under the following conditions: DMS temperature low, modifier (propanol) composition low, separation voltage 3500 V, DMS resolution enhancement low. For method #2, the DMS cell was not activated. The MS operated under the following conditions: curtain gas 17, CAD gas medium, ion spray voltage 4100 V in ESI + mode and −2500 V in ESI− mode, temperature 200 °C, nebulizing gas 17, and heater gas 25. First, PC, PE, (L)PC, (L)PE, and SM lipid classes were analyzed. Next, FFA, TAG, DAG, CER, dihydroceramide (DCER), lactosylceramide (LCER), hexosylceramide (HCER), and CE lipids were analyzed applying method #2. Further technical detail including a list of all monitored transitions and detailed experimental setting can be found elsewhere [24,25,26]. In the erythrocyte samples, lipid concentrations were corrected for the erythrocyte protein pellet content, which was quantified using a Micro BCA Protein Assay Kit (Thermo Scientific, Waltham, MA, USA). Samples were measured in a randomized batch-controlled fashion. The plasma samples were measured in 2 consecutive batches and the erythrocyte samples were spread across 4 consecutive batches, with baseline and follow-up samples of each patient included in the same batch. Four quality controls (QC) consisting of a commercial freeze-dried plasma reference were added to each measurement batch. For both plasma and erythrocyte measurements, we used QCs consisting of pooled reference plasma.

### 4.3. Data Processing

Data from both sample types were pre-processed in the same standardized manner. Lipid species were excluded from further analysis if the relative standard deviation (RSD) of the QCs was >20% within each batch or >25% between batches. Approximately 10% of the detected lipids in erythrocytes were not detected in the QCs, hampering exclusion based on the RSD. However, these lipids were also not detected in most of the patient samples, and therefore automatically excluded in our last processing step in which we excluded lipid species that were not detected in >75% of observations or when they were observed only in a single batch. Percentage missing values was 3% and 18% in plasma and erythrocyte measurements, respectively. All remaining missing observations in plasma and erythrocyte samples were imputed with the minimum measured value divided by 2. As a measure of technical variation, the RSDs of the QCs of the lipid classes, containing the lipid species included in the analyses, are provided in Table S1.

### 4.4. Statistical Analyses

Prior to the analyses, all lipid variables were logarithmically transformed due to a non-normal distribution. We calculated the intra-class correlation coefficients (ICC) and corresponding 95% confidence interval (CI) for each lipid, separately for plasma and erythrocyte samples. We fitted linear mixed-effects models with restricted maximum likelihood, including patients as random effects (random-intercept) (Stata command: mixed ‘lipid’ time || patient ID:, reml var). For each lipid, ICCs were calculated as the ratio of the between-subject variance to the total variance composed of the sum of between- and within-subject variance using the postestimation command estat icc in Stata. The reproducibility was categorized on the basis of the ICCs as follows: excellent ≥0.80, good <0.80–≥0.60, moderate >0.60–≥0.40, and poor <0.40. The ICC score distributions were compared between sample types by two-sided paired signed-ranks Wilcoxon tests with exact probabilities (command: signrank). Stata V16.1 (StataCorp LP, College Station, TX, USA) was used for all analyses.

### 4.5. Data Availability and Lipid Nomenclature

The data underlying this article cannot be shared publicly due to the privacy of the participants of the HOPE study and legal reasons (HOPE study participants did not sign informed consent to make their data publicly available). The data are available upon request to interested qualified researchers. Data requests should be sent to the corresponding author. As the Lipidyzer is not capable of specifying the exact sn-position of the FA side chains, we adopted the lipid short-hand notation as described by Liebisch et al. [27]. Additionally, it has to be noted that for TAG lipids, the Lipidyzer platform is capable of defining one of the three FA side chains. Hence, a TAG lipid specified as for example TG 54:6-FA 18:1 would refer to a TAG lipid with 54 carbons, 6 double bonds, and 1 side chain being FA 18:1.

## Figures and Tables

**Table 1:** Number of individual lipids per class and class concentrations in plasma and erythrocytes.

	Plasma		Erythrocytes	
	Number of Lipid Species	Class Concentration (nmol/mL)	Number of Lipid Species	Class Concentration (nmol/mL)
Triacylglycerols	482	1579.4 (1064.9–3195.2)	134	6.5 (5.6–9.4)
Diacylglycerols	9	13.3 (8.4–22.2)	10	5.8 (4.7–6.2)
Free fatty acids	20	745.3 (552.0–1202.9)	20	486.9 (379.2–669.2)
Cholesteryl esters	24	4571.6 (4065.1–5521.3)	5	1.2 (0.9–1.7)
Phosphatidylcholines	31	4013.7 (3203.1–4661.6)	42	3899.2 (3723.0–4296.6)
Phosphatidylethanolamines	26	156.2 (120.9–180.3)	42	3954.6 (3721.9–4323.3)
Lysophosphatidylcholines	9	385.9 (335.6–442.9)	7	119.8 (109.7–168.9)
Lysophosphatidylethanolamines	2	4.2 (3.5–4.9)	4	8.6 (6.8–9.7)
Sphingomyelins	12	1204.6 (1037.0–1351.9)	8	2695.8 (2434.8–2815.6)
Ceramides	6	14.1 (11.9–17.4)	7	163.0 (133.3–186.4)
Dihydroceramides	2	1.0 (0.8–1.3)	1	1.8 (1.4–2.1)
Hexosylceramides	5	5.1 (4.7–5.9)	4	5.6 (5.0–7.4)
Lactosylceramides	2	3.4 (2.7–3.8)	2	23.8 (20.6–33.5)

Numbers represent median (interquartile range) unless otherwise specified. Data represent baseline measurements.

## Data Availability

The data presented in this study are available on request from the corresponding author. The data are not publicly available due to the privacy of the participants of the HOPE study and legal reasons (HOPE study participants did not sign informed consent to make their data publicly available).

## Supplementary Materials

The following are available online at https://www.mdpi.com/2218-1989/11/1/26/s1: Table S1: Technical variability of quality controls, stratified by lipid class, Table S2: Intra-class correlation coefficients of lipid levels between baseline and week 6 in plasma and erythrocytes, Table S3: Reproducibility of lipids in plasma and erythrocytes, stratified by lipid class.
