# Supplementary material for: Reproducibility of Targeted Lipidome Analyses (Lipidyzer) in Plasma and Erythrocytes over a 6-Week Period
Source: Metabolites. 2020 Dec 31;11(1):26. doi: 10.3390/metabo11010026 (PMC7823270; doi:10.3390/metabo11010026)
Supplement: Supplementary file 1 [file metabolites-11-00026-s001.pdf]

SUPPLEMENTARY FILE

Table S1. Technical variability of quality controls, stratified by lipid class

	Lipids in analyses	
	Plasma batches	Erythrocyte batches
Cholesteryl esters	0.13	0.12
Ceramides	0.09	0.19
Diacylglycerols	0.11	0.14
Dihydroceramides	0.21	0.16
Free fatty acids	0.08	0.13
Hexosylceramides	0.12	0.15
Lactosylceramides	0.06	0.16
Lysophosphatidylcholines	0.15	0.13
Lysophosphatidylethanolamines	0.14	0.14
Phosphatidylcholines	0.03	0.12
Phosphatidylethanolamines	0.11	0.13
Sphingomyelins	0.05	0.10
Triacylglycerols	0.09	0.12

Numbers represent the relative standard deviations of the included quality controls, separate for the batches in which plasma and erythrocytes were measured.

Table S2. Intra-class correlation coefficients of lipid levels between baseline and week 6 in plasma and erythrocytes

Lipid	Plasma			Erythrocytes		
	ICC	95% CI	Δ (%)	ICC	95% CI	Δ (%)
**CE 12:0**	0.45	0.19 0.74	-0.58%			
**CE 14:0**	0.64	0.42 0.82	-0.94%			
**CE 14:1**	0.76	0.58 0.88	-0.12%			
**CE 15:0**	0.54	0.30 0.77	-0.38%			
**CE 16:0**	0.60	0.36 0.80	-0.27%	0.52	0.22 0.81	35.96%
**CE 16:1**	0.79	0.62 0.89	-1.38%	0.61	0.34 0.83	16.52%
**CE 17:0**	0.65	0.43 0.82	-1.26%			
**CE 18:0**	0.63	0.40 0.81	0.92%			
**CE 18:1**	0.65	0.42 0.82	-0.58%	0.23	0.02 0.82	61.30%
**CE 18:2**	0.74	0.55 0.87	-1.18%	0.19	0.02 0.77	84.02%
**CE 18:3**	0.77	0.59 0.89	2.03%			
**CE 18:4**	0.78	0.59 0.89	7.18%			
**CE 20:0**	0.18	0.02 0.69	-8.38%			
**CE 20:1**	0.64	0.41 0.82	-3.64%			
**CE 20:2**	0.64	0.42 0.82	-0.24%			
**CE 20:3**	0.63	0.40 0.81	-0.25%			
**CE 20:4**	0.72	0.52 0.85	-3.43%	0.35	0.11 0.70	116.67%
**CE 20:5**	0.78	0.60 0.89	-2.76%			
**CE 22:0**	0.57	0.33 0.79	4.15%			
**CE 22:1**	0.56	0.31 0.78	3.03%			
**CE 22:4**	0.53	0.29 0.76	-6.13%			
**CE 22:5**	0.58	0.34 0.78	-3.06%			
**CE 22:6**	0.73	0.53 0.86	-7.39%			
**CE 24:0**	0.22	0.04 0.67	11.69%			
**Cer d18:1/14:0**				0.33	0.11 0.67	-1.44%
**Cer d18:1/16:0**	0.47	0.22 0.74	1.34%	0.58	0.33 0.79	3.00%
**Cer d18:1/18:0**	0.48	0.23 0.74	-4.91%			
**Cer d18:1/20:0**	0.21	0.03 0.70	1.18%	0.76	0.58 0.88	6.44%
**Cer d18:1/22:0**	0.71	0.49 0.86	0.05%	0.88	0.78 0.94	5.65%
**Cer d18:1/24:0**	0.79	0.62 0.90	-0.23%	0.89	0.79 0.94	5.86%
**Cer d18:1/24:1**	0.64	0.42 0.81	-3.81%	0.81	0.65 0.91	4.63%
**Cer d18:1/26:0**				0.87	0.76 0.94	4.48%
**DG 12:0_18:1**	0.00	0.00 0.00	11.03%			
**DG 14:0_16:0**	0.22	0.04 0.69	79.96%			
**DG 16:0_16:0**	0.32	0.09 0.69	31.31%	0.62	0.37 0.82	7.96%
**DG 16:0_16:1**	0.56	0.31 0.78	3.90%			
**DG 16:0_18:1**	0.48	0.22 0.75	-1.23%	0.78	0.60 0.89	8.89%
**DG 16:0_18:2**				0.72	0.51 0.87	3.69%
**DG 16:0_20:4**	0.00	0.00 0.00	23.03%			
**DG 16:0_22:5**				0.19	0.02 0.71	17.38%
**DG 16:1_18:1**	0.62	0.39 0.81	-7.84%			
**DG 16:1_20:0**				0.05	0.00 0.99	-4.46%
**DG 18:0_18:1**				0.35	0.12 0.68	3.15%
**DG 18:0_18:2**				0.66	0.44 0.83	7.85%
**DG 18:1_18:1**	0.58	0.33 0.80	-8.43%	0.46	0.19 0.75	11.21%
**DG 18:1_18:2**				0.52	0.27 0.76	-6.60%
**DG 18:1_20:4**				0.38	0.14 0.69	5.35%

**DG 18:1_22:6**	0.34	0.10	0.71	-23.44%				
**Cer d18:1/22:0**					0.71	0.51	0.86	7.16%
**Cer d18:1/24:0**	0.58	0.33	0.80	10.34%				
**Cer d18:1/24:1**	0.03	0.00	1.00	-9.32%				
**FFA 12:0**	0.65	0.42	0.82	6.17%	0.66	0.42	0.85	2.40%
**FFA 14:0**	0.69	0.47	0.85	4.48%	0.82	0.65	0.92	3.04%
**FFA 14:1**	0.57	0.31	0.79	-2.70%				
**FFA 15:0**	0.54	0.27	0.78	5.22%				
**FFA 16:0**	0.68	0.46	0.84	-4.24%	0.29	0.07	0.70	16.85%
**FFA 16:1**	0.65	0.42	0.83	-9.79%	0.79	0.59	0.91	-0.67%
**FFA 17:0**	0.74	0.54	0.87	0.20%				
**FFA 18:0**	0.59	0.35	0.79	-7.52%	0.31	0.08	0.69	16.73%
**FFA 18:1**	0.65	0.42	0.83	-9.15%	0.71	0.47	0.87	15.87%
**FFA 18:2**	0.56	0.30	0.79	-7.57%	0.73	0.51	0.88	18.34%
**FFA 18:3**	0.62	0.37	0.82	-7.39%	0.58	0.33	0.79	6.36%
**FFA 18:4**	0.54	0.29	0.76	-7.41%	0.17	0.01	0.78	6.90%
**FFA 20:1**	0.79	0.62	0.90	-5.10%	0.47	0.16	0.81	2.77%
**FFA 20:2**	0.68	0.47	0.84	-2.86%	0.75	0.51	0.89	1.46%
**FFA 20:3**	0.71	0.50	0.85	-1.04%	0.36	0.11	0.71	5.70%
**FFA 20:4**	0.68	0.46	0.84	-4.56%	0.69	0.48	0.84	12.58%
**FFA 20:5**	0.60	0.37	0.79	0.61%	0.52	0.28	0.75	4.80%
**FFA 22:1**					0.25	0.05	0.69	15.57%
**FFA 22:2**					0.69	0.41	0.88	0.18%
**FFA 22:4**	0.74	0.54	0.87	-0.05%	0.14	0.01	0.77	0.26%
**FFA 22:5**	0.52	0.27	0.77	4.45%	0.32	0.10	0.66	1.48%
**FFA 22:6**	0.64	0.43	0.81	-2.56%	0.38	0.14	0.69	7.49%
**FFA 24:0**					0.77	0.56	0.90	1.54%
**HexCer d18:1/16:0**	0.50	0.25	0.75	-1.97%	0.91	0.83	0.96	4.59%
**HexCer d18:1/20:0**	0.10	0.00	0.88	7.79%				
**HexCer d18:1/22:0**	0.66	0.43	0.83	0.57%	0.44	0.19	0.73	4.82%
**HexCer d18:1/24:0**	0.64	0.41	0.82	0.78%	0.72	0.51	0.86	6.85%
**HexCer d18:1/24:1**	0.58	0.34	0.79	-2.08%	0.71	0.50	0.85	5.25%
**LacCer d18:1/16:0**	0.78	0.61	0.89	-0.83%	0.44	0.19	0.72	6.81%
**LacCer d18:1/24:0**					0.75	0.57	0.88	1.12%
**LacCer d18:1/24:1**	0.54	0.30	0.76	-4.09%				
**LPC 15:0**	0.36	0.12	0.70	13.01%				
**LPC 16:0**	0.44	0.21	0.71	2.74%	0.69	0.47	0.85	7.22%
**LPC 17:0**	0.38	0.14	0.70	-0.24%	0.24	0.04	0.70	9.91%
**LPC 18:0**	0.57	0.33	0.78	4.86%	0.74	0.55	0.87	5.15%
**LPC 18:1**	0.56	0.33	0.77	3.82%	0.70	0.49	0.85	11.57%
**LPC 18:2**	0.65	0.42	0.82	1.98%	0.86	0.74	0.93	10.79%
**LPC 20:2**	0.15	0.01	0.78	26.81%				
**LPC 20:3**	0.65	0.43	0.82	5.89%	0.24	0.05	0.66	23.65%
**LPC 20:4**	0.73	0.54	0.86	-4.72%	0.66	0.44	0.82	-0.05%
**LPE 16:0**	0.46	0.22	0.73	2.73%	0.87	0.76	0.94	5.84%
**LPE 18:0**					0.74	0.54	0.87	8.79%
**LPE 18:1**	0.49	0.24	0.75	3.82%				
**LPE 20:3**					0.73	0.54	0.86	4.54%
**LPE 22:5**					0.55	0.31	0.77	-6.26%
**PC 14:0_18:2**	0.78	0.60	0.89	-0.25%	0.71	0.50	0.85	-2.27%
**PC 14:0_20:3**					0.44	0.19	0.73	-0.80%

**PC 15:0_18:1**				0.45	0.21 0.72	-0.11%
**PC 15:0_18:2**	0.53	0.28 0.77	-0.92%	0.38	0.13 0.73	-2.00%
**PC 16:0_14:0**				0.79	0.63 0.89	2.45%
**PC 16:0_16:0**	0.70	0.49 0.85	2.67%	0.48	0.24 0.74	3.05%
**PC 16:0_16:1**	0.79	0.62 0.89	5.22%	0.87	0.76 0.94	4.83%
**PC 16:0_18:0**	0.76	0.58 0.88	-2.37%	0.59	0.36 0.79	4.27%
**PC 16:0_18:1**	0.70	0.50 0.85	-1.56%	0.84	0.71 0.92	5.71%
**PC 16:0_18:2**	0.64	0.39 0.83	1.42%	0.79	0.62 0.89	2.70%
**PC 16:0_18:3**	0.64	0.41 0.82	1.56%	0.77	0.60 0.89	5.75%
**PC 16:0_20:1**				0.60	0.35 0.81	5.00%
**PC 16:0_20:2**	0.46	0.20 0.74	0.97%	0.74	0.54 0.88	4.92%
**PC 16:0_20:3**	0.58	0.34 0.79	-1.66%	0.71	0.52 0.85	6.97%
**PC 16:0_20:4**	0.71	0.52 0.85	-6.59%	0.77	0.59 0.88	6.47%
**PC 16:0_20:5**	0.73	0.52 0.87	2.83%	0.75	0.57 0.87	8.63%
**PC 16:0_22:4**				0.73	0.54 0.86	10.38%
**PC 16:0_22:5**	0.66	0.44 0.83	5.47%	0.46	0.22 0.71	3.66%
**PC 16:0_22:6**	0.70	0.49 0.85	-5.64%	0.66	0.43 0.83	-2.06%
**PC 17:0_18:1**	0.63	0.40 0.82	5.87%	0.39	0.14 0.70	-1.52%
**PC 17:0_18:2**	0.58	0.32 0.80	-1.23%	0.52	0.27 0.76	-2.10%
**PC 18:0_16:1**	0.70	0.50 0.85	1.23%			
**PC 18:0_18:0**				0.56	0.30 0.79	4.40%
**PC 18:0_18:1**	0.66	0.44 0.83	-0.51%	0.76	0.57 0.88	7.04%
**PC 18:0_18:2**	0.64	0.40 0.83	2.54%	0.78	0.60 0.89	2.86%
**PC 18:0_18:3**				0.73	0.54 0.86	3.74%
**PC 18:0_20:2**	0.50	0.26 0.74	-6.03%			
**PC 18:0_20:3**	0.67	0.45 0.83	1.08%	0.76	0.57 0.88	3.92%
**PC 18:0_20:4**	0.73	0.54 0.86	-5.08%	0.77	0.59 0.89	5.91%
**PC 18:0_20:5**				0.73	0.54 0.86	6.24%
**PC 18:0_22:5**				0.82	0.67 0.91	0.11%
**PC 18:0_22:6**	0.69	0.48 0.85	-2.74%	0.82	0.66 0.91	-0.40%
**PC 18:1_16:1**	0.59	0.35 0.79	-7.47%	0.39	0.16 0.68	1.36%
**PC 18:1_18:1**	0.58	0.35 0.79	-7.83%	0.73	0.54 0.86	6.00%
**PC 18:1_18:2**	0.63	0.40 0.82	-2.47%	0.82	0.67 0.91	0.92%
**PC 18:1_18:3**				0.48	0.22 0.75	8.56%
**PC 18:1_20:3**	0.44	0.20 0.72	-5.91%	0.37	0.14 0.68	3.49%
**PC 18:1_20:4**	0.68	0.47 0.83	-12.25%	0.72	0.51 0.86	3.16%
**PC 18:1_20:5**				0.26	0.05 0.68	-6.50%
**PC 18:1_22:6**				0.19	0.02 0.70	8.34%
**PC 18:2_16:1**	0.71	0.50 0.86	1.46%	0.45	0.21 0.72	-2.72%
**PC 18:2_18:2**	0.77	0.58 0.89	3.60%	0.50	0.23 0.76	-4.00%
**PC 18:2_20:3**				0.44	0.16 0.76	1.09%
**PC 18:2_20:4**	0.59	0.35 0.80	-5.41%	0.58	0.33 0.80	2.45%
**PE 16:0_18:1**	0.53	0.27 0.77	9.46%	0.80	0.65 0.90	1.32%
**PE 16:0_18:2**	0.43	0.17 0.73	8.47%	0.77	0.59 0.88	-0.23%
**PE 16:0_20:4**	0.53	0.28 0.77	1.74%	0.63	0.39 0.81	7.02%
**PE 16:0_22:6**	0.23	0.04 0.70	20.85%			
**PE 18:0_18:0**	0.57	0.31 0.79	7.58%			
**PE 18:0_18:1**	0.40	0.15 0.72	2.32%			
**PE 18:0_18:2**	0.51	0.25 0.77	3.32%			
**PE 18:0_20:4**	0.56	0.32 0.78	-8.05%	0.46	0.21 0.73	9.11%
**PE 18:1_16:1**				0.88	0.78 0.94	4.49%

**PE 18:1_18:1**	0.40	0.15 0.72	7.98%	0.88	0.77 0.94	1.57%
**PE 18:1_18:2**	0.46	0.20 0.74	10.01%	0.65	0.44 0.82	0.54%
**PE 18:1_20:4**	0.43	0.17 0.73	-9.64%			
**PE 18:1_22:5**				0.08	0.00 0.96	3.27%
**PE 18:1_22:6**				0.57	0.32 0.79	-3.56%
**PE 18:2_16:1**				0.80	0.64 0.90	3.74%
**PE 18:2_18:2**	0.52	0.26 0.77	9.72%			
**PE 18:2_20:4**				0.71	0.51 0.86	3.24%
**PE O-16:0_18:1**				0.77	0.60 0.88	5.85%
**PE O-16:0_18:2**				0.75	0.56 0.88	8.90%
**PE O-16:0_20:3**				0.40	0.15 0.71	-4.24%
**PE O-16:0_20:4**				0.73	0.54 0.86	8.26%
**PE O-16:0_22:4**				0.65	0.42 0.83	10.11%
**PE O-16:0_22:5**				0.17	0.01 0.75	8.84%
**PE O-18:0_18:2**	0.49	0.24 0.75	11.00%			
**PE O-18:0_20:3**				0.34	0.11 0.69	-9.45%
**PE O-18:0_20:4**				0.74	0.55 0.87	7.23%
**PE O-18:0_22:4**				0.59	0.35 0.80	9.41%
**PE P-16:0_18:0**				0.39	0.15 0.69	0.16%
**PE P-16:0_18:1**	0.57	0.33 0.79	11.48%	0.68	0.47 0.84	3.22%
**PE P-16:0_18:2**	0.62	0.39 0.81	17.56%	0.77	0.59 0.89	7.03%
**PE P-16:0_20:3**	0.29	0.08 0.66	28.50%	0.34	0.11 0.70	-5.90%
**PE P-16:0_20:4**	0.35	0.12 0.69	8.91%	0.50	0.24 0.76	5.51%
**PE P-16:0_22:4**				0.70	0.49 0.85	8.20%
**PE P-16:0_22:5**				0.01	0.00 1.00	6.35%
**PE P-16:0_22:6**	0.15	0.01 0.75	39.17%			
**PE P-18:0_16:0**				0.28	0.08 0.66	2.18%
**PE P-18:0_18:1**	0.72	0.52 0.86	10.58%	0.72	0.52 0.86	2.73%
**PE P-18:0_18:2**	0.61	0.37 0.81	10.65%	0.72	0.53 0.86	7.82%
**PE P-18:0_20:3**				0.43	0.18 0.73	-5.51%
**PE P-18:0_20:4**	0.51	0.25 0.76	4.61%	0.52	0.26 0.76	7.40%
**PE P-18:0_22:4**				0.59	0.33 0.81	8.09%
**PE P-18:0_22:5**				0.08	0.00 0.95	6.03%
**PE P-18:0_22:6**	0.18	0.02 0.73	42.40%	0.63	0.39 0.82	-2.47%
**PE P-18:1_18:1**	0.71	0.51 0.86	2.72%	0.74	0.55 0.87	2.03%
**PE P-18:1_18:2**	0.60	0.36 0.80	6.36%	0.83	0.68 0.92	4.50%
**PE P-18:1_20:3**				0.28	0.06 0.70	-6.83%
**PE P-18:1_20:4**	0.36	0.12 0.69	0.38%	0.64	0.40 0.83	6.51%
**PE P-18:1_20:5**				0.56	0.31 0.78	2.50%
**PE P-18:1_22:5**				0.00	0.00 0.00	2.31%
**PE P-18:1_22:6**				0.52	0.25 0.77	-3.90%
**PE P-18:2_20:4**	0.00	0.00 0.00	14.44%			
**SM 14:0**	0.76	0.58 0.88	-0.21%	0.91	0.82 0.95	4.82%
**SM 16:0**	0.67	0.45 0.83	-1.17%	0.75	0.55 0.88	5.39%
**SM 18:0**	0.76	0.58 0.88	-1.56%	0.82	0.67 0.91	1.68%
**SM 18:1**	0.73	0.53 0.86	-3.33%	0.87	0.76 0.94	3.24%
**SM 20:0**	0.76	0.57 0.88	2.03%			
**SM 20:1**	0.73	0.53 0.87	4.19%			
**SM 22:0**	0.74	0.54 0.87	1.04%			
**SM 22:1**	0.65	0.42 0.83	1.74%			
**SM 24:0**	0.93	0.86 0.96	0.82%	0.82	0.68 0.91	3.26%

**SM 24:1**	0.82	0.66	0.91	1.88%	0.88	0.78	0.94	3.30%
**SM 26:0**	0.79	0.62	0.89	1.19%	0.91	0.83	0.96	2.10%
**SM 26:1**	0.86	0.74	0.93	1.93%	0.93	0.86	0.96	1.38%
**TG 36:0-FA 12:0**	0.30	0.08	0.68	-9.42%				
**TG 40:0-FA 12:0**	0.30	0.08	0.68	25.01%				
**TG 40:0-FA 14:0**	0.28	0.07	0.68	47.93%				
**TG 42:0-FA 12:0**	0.44	0.19	0.72	47.57%				
**TG 42:0-FA 14:0**	0.35	0.12	0.69	46.39%				
**TG 42:0-FA 16:0**	0.41	0.16	0.71	50.04%	0.16	0.01	0.76	-10.49%
**TG 42:1-FA 12:0**	0.43	0.19	0.72	53.55%				
**TG 42:1-FA 14:0**	0.38	0.14	0.70	55.54%				
**TG 42:1-FA 16:0**	0.51	0.25	0.75	55.06%				
**TG 42:1-FA 16:1**	0.38	0.14	0.70	53.85%				
**TG 42:1-FA 18:1**	0.40	0.16	0.71	56.94%				
**TG 42:2-FA 12:0**	0.59	0.35	0.79	82.50%				
**TG 42:2-FA 18:2**	0.44	0.19	0.72	70.07%				
**TG 44:0-FA 12:0**	0.47	0.22	0.73	49.36%				
**TG 44:0-FA 14:0**	0.47	0.22	0.74	39.19%	0.39	0.12	0.75	-4.18%
**TG 44:0-FA 16:0**	0.47	0.22	0.74	45.26%	0.23	0.03	0.72	0.09%
**TG 44:0-FA 18:0**	0.32	0.09	0.69	47.77%				
**TG 44:1-FA 12:0**	0.52	0.27	0.75	54.45%				
**TG 44:1-FA 14:0**	0.52	0.28	0.76	48.83%	0.67	0.44	0.84	5.59%
**TG 44:1-FA 16:0**	0.54	0.30	0.77	44.47%				
**TG 44:1-FA 16:1**	0.62	0.39	0.81	37.00%	0.46	0.19	0.76	0.81%
**TG 44:1-FA 18:1**	0.44	0.19	0.72	54.21%	0.36	0.13	0.69	17.56%
**TG 44:2-FA 12:0**	0.57	0.33	0.78	59.74%				
**TG 44:2-FA 14:0**	0.55	0.30	0.77	58.87%				
**TG 44:2-FA 16:0**	0.56	0.31	0.78	44.31%				
**TG 44:2-FA 16:1**	0.57	0.33	0.78	39.16%				
**TG 44:2-FA 18:1**	0.48	0.23	0.74	50.10%				
**TG 44:2-FA 18:2**	0.53	0.28	0.77	55.29%				
**TG 44:3-FA 18:2**	0.57	0.32	0.79	49.10%				
**TG 45:0-FA 14:0**	0.46	0.22	0.73	50.26%				
**TG 45:0-FA 15:0**	0.44	0.20	0.72	42.75%	0.37	0.12	0.73	-1.04%
**TG 45:0-FA 16:0**					0.46	0.19	0.77	-5.81%
**TG 45:1-FA 15:0**	0.45	0.21	0.72	53.18%				
**TG 45:1-FA 16:0**	0.51	0.26	0.75	37.28%				
**TG 45:1-FA 18:1**	0.40	0.15	0.70	61.61%				
**TG 46:0-FA 12:0**	0.50	0.25	0.75	42.02%				
**TG 46:0-FA 14:0**	0.56	0.31	0.78	32.02%	0.23	0.03	0.78	1.05%
**TG 46:0-FA 16:0**	0.58	0.34	0.79	32.40%	0.47	0.18	0.78	1.20%
**TG 46:0-FA 18:0**	0.44	0.20	0.72	43.33%				
**TG 46:1-FA 12:0**	0.59	0.35	0.79	43.17%				
**TG 46:1-FA 14:0**	0.64	0.42	0.82	29.98%	0.36	0.10	0.73	-1.68%
**TG 46:1-FA 14:1**	0.64	0.41	0.82	26.86%				
**TG 46:1-FA 16:0**	0.64	0.41	0.82	33.53%	0.64	0.39	0.83	5.68%
**TG 46:1-FA 16:1**	0.71	0.51	0.85	19.28%	0.76	0.56	0.88	7.61%
**TG 46:1-FA 18:0**	0.48	0.22	0.74	37.81%				
**TG 46:1-FA 18:1**	0.58	0.34	0.79	43.07%	0.56	0.30	0.79	17.56%
**TG 46:2-FA 12:0**	0.65	0.43	0.82	37.36%				
**TG 46:2-FA 14:0**	0.63	0.41	0.81	31.22%	0.21	0.02	0.80	7.11%

**TG 46:2-FA 14:1**	0.66	0.44 0.83	25.27%			
**TG 46:2-FA 16:0**	0.64	0.42 0.82	36.27%			
**TG 46:2-FA 16:1**	0.68	0.48 0.84	19.09%	0.65	0.39 0.84	6.94%
**TG 46:2-FA 18:1**	0.54	0.29 0.77	32.01%	0.59	0.34 0.80	9.88%
**TG 46:2-FA 18:2**	0.63	0.40 0.81	39.18%			
**TG 46:3-FA 12:0**	0.66	0.44 0.83	37.67%			
**TG 46:3-FA 14:0**	0.67	0.45 0.83	36.78%			
**TG 46:3-FA 14:1**	0.57	0.34 0.78	28.12%			
**TG 46:3-FA 16:0**	0.67	0.45 0.83	40.58%			
**TG 46:3-FA 16:1**	0.60	0.37 0.80	25.01%			
**TG 46:3-FA 18:1**	0.52	0.26 0.77	36.33%			
**TG 46:3-FA 18:2**	0.60	0.36 0.80	34.38%			
**TG 46:3-FA 18:3**	0.67	0.45 0.83	43.64%			
**TG 46:4-FA 18:2**	0.53	0.27 0.77	28.63%			
**TG 47:0-FA 14:0**	0.52	0.27 0.76	42.37%	0.52	0.23 0.79	-3.52%
**TG 47:0-FA 15:0**	0.55	0.31 0.78	36.28%	0.35	0.09 0.74	1.23%
**TG 47:0-FA 16:0**	0.54	0.29 0.77	37.40%	0.56	0.27 0.81	2.94%
**TG 47:0-FA 17:0**	0.51	0.26 0.75	39.59%	0.59	0.32 0.81	8.12%
**TG 47:1-FA 14:0**	0.58	0.35 0.79	36.00%	0.31	0.07 0.74	1.66%
**TG 47:1-FA 15:0**	0.63	0.40 0.81	30.94%	0.60	0.35 0.81	2.35%
**TG 47:1-FA 16:0**	0.66	0.45 0.83	24.12%	0.52	0.24 0.78	5.11%
**TG 47:1-FA 16:1**	0.58	0.34 0.78	17.60%			
**TG 47:1-FA 17:0**	0.57	0.33 0.78	41.13%			
**TG 47:1-FA 18:1**	0.59	0.35 0.79	38.31%			
**TG 47:2-FA 14:0**	0.62	0.39 0.81	31.30%			
**TG 47:2-FA 15:0**	0.66	0.44 0.83	29.82%			
**TG 47:2-FA 16:1**	0.58	0.35 0.78	19.74%			
**TG 47:2-FA 18:1**	0.56	0.31 0.78	27.39%	0.58	0.32 0.80	5.73%
**TG 47:2-FA 18:2**	0.62	0.39 0.81	39.76%			
**TG 48:0-FA 14:0**	0.62	0.39 0.81	25.75%			
**TG 48:0-FA 16:0**	0.62	0.39 0.81	21.88%	0.27	0.05 0.75	21.86%
**TG 48:0-FA 18:0**	0.59	0.35 0.79	28.82%			
**TG 48:1-FA 12:0**	0.58	0.34 0.79	32.51%			
**TG 48:1-FA 14:0**	0.71	0.50 0.85	21.56%	0.47	0.19 0.76	35.70%
**TG 48:1-FA 14:1**	0.69	0.48 0.84	19.51%			
**TG 48:1-FA 16:0**	0.73	0.53 0.86	16.82%	0.63	0.36 0.84	12.82%
**TG 48:1-FA 16:1**	0.76	0.58 0.88	10.88%	0.50	0.23 0.78	1.03%
**TG 48:1-FA 18:0**	0.60	0.36 0.80	29.07%			
**TG 48:1-FA 18:1**	0.70	0.50 0.85	21.17%	0.47	0.21 0.75	22.91%
**TG 48:2-FA 12:0**	0.62	0.39 0.81	29.61%			
**TG 48:2-FA 14:0**	0.73	0.54 0.87	12.40%	0.43	0.16 0.75	17.57%
**TG 48:2-FA 14:1**	0.70	0.50 0.85	16.22%			
**TG 48:2-FA 16:0**	0.73	0.54 0.87	14.28%	0.55	0.28 0.80	8.94%
**TG 48:2-FA 16:1**	0.77	0.60 0.88	5.35%	0.62	0.36 0.83	2.22%
**TG 48:2-FA 18:0**	0.64	0.41 0.82	20.36%			
**TG 48:2-FA 18:1**	0.67	0.46 0.83	20.82%	0.36	0.10 0.74	12.45%
**TG 48:2-FA 18:2**	0.75	0.56 0.87	18.19%			
**TG 48:3-FA 12:0**	0.67	0.46 0.84	29.46%			
**TG 48:3-FA 14:0**	0.77	0.59 0.88	10.27%			
**TG 48:3-FA 14:1**	0.73	0.53 0.86	10.59%			
**TG 48:3-FA 16:0**	0.75	0.57 0.88	16.71%			

**TG 48:3-FA 16:1**	0.73	0.54 0.86	5.71%	0.69	0.46 0.86	4.83%
**TG 48:3-FA 18:1**	0.67	0.45 0.83	24.88%			
**TG 48:3-FA 18:2**	0.73	0.54 0.87	19.55%			
**TG 48:3-FA 18:3**	0.73	0.54 0.87	22.05%			
**TG 48:4-FA 12:0**	0.70	0.49 0.85	28.59%			
**TG 48:4-FA 14:0**	0.71	0.50 0.85	20.42%			
**TG 48:4-FA 14:1**	0.71	0.50 0.85	10.47%			
**TG 48:4-FA 16:0**	0.72	0.52 0.86	24.96%			
**TG 48:4-FA 16:1**	0.68	0.47 0.84	13.51%			
**TG 48:4-FA 18:1**	0.66	0.43 0.83	32.19%			
**TG 48:4-FA 18:2**	0.72	0.52 0.86	18.66%			
**TG 48:4-FA 18:3**	0.74	0.55 0.87	24.18%			
**TG 48:5-FA 18:2**	0.64	0.41 0.82	26.48%			
**TG 48:5-FA 18:3**	0.67	0.45 0.84	29.64%			
**TG 49:0-FA 15:0**	0.65	0.43 0.82	25.09%			
**TG 49:0-FA 16:0**	0.60	0.36 0.80	29.00%	0.29	0.04 0.81	-4.62%
**TG 49:0-FA 17:0**	0.56	0.32 0.78	31.22%	0.30	0.06 0.75	11.96%
**TG 49:0-FA 18:0**	0.53	0.28 0.77	32.88%			
**TG 49:1-FA 14:0**	0.64	0.41 0.82	31.16%			
**TG 49:1-FA 15:0**	0.71	0.51 0.85	18.69%	0.52	0.26 0.78	11.84%
**TG 49:1-FA 16:0**	0.71	0.50 0.85	17.96%	0.66	0.42 0.84	7.89%
**TG 49:1-FA 16:1**	0.70	0.50 0.85	17.13%	0.47	0.19 0.77	14.96%
**TG 49:1-FA 17:0**	0.68	0.46 0.84	24.32%	0.64	0.39 0.83	9.25%
**TG 49:1-FA 18:1**	0.69	0.48 0.84	21.55%	0.63	0.38 0.82	17.26%
**TG 49:2-FA 14:0**	0.68	0.47 0.84	17.50%			
**TG 49:2-FA 15:0**	0.75	0.57 0.88	9.59%	0.62	0.36 0.82	7.38%
**TG 49:2-FA 16:0**	0.75	0.57 0.87	10.90%	0.48	0.20 0.77	6.49%
**TG 49:2-FA 16:1**	0.73	0.54 0.86	8.27%	0.77	0.58 0.89	8.41%
**TG 49:2-FA 17:0**	0.71	0.50 0.85	20.86%			
**TG 49:2-FA 18:1**	0.71	0.51 0.85	11.34%	0.42	0.15 0.75	11.93%
**TG 49:2-FA 18:2**	0.75	0.56 0.87	17.33%			
**TG 49:3-FA 15:0**	0.80	0.64 0.90	6.62%			
**TG 49:3-FA 16:0**	0.76	0.57 0.88	11.98%			
**TG 49:3-FA 16:1**	0.75	0.56 0.87	4.09%	0.60	0.33 0.82	8.20%
**TG 49:3-FA 18:2**	0.76	0.59 0.88	7.12%			
**TG 49:3-FA 18:3**	0.74	0.54 0.87	16.28%			
**TG 50:0-FA 14:0**	0.60	0.36 0.80	24.65%			
**TG 50:0-FA 16:0**	0.70	0.49 0.85	13.32%	0.15	0.01 0.78	64.40%
**TG 50:0-FA 18:0**	0.66	0.43 0.83	15.29%	0.14	0.01 0.78	20.36%
**TG 50:1-FA 14:0**	0.70	0.49 0.85	15.99%			
**TG 50:1-FA 16:0**	0.76	0.58 0.88	10.77%	0.20	0.03 0.70	257.87%
**TG 50:1-FA 16:1**	0.78	0.61 0.89	5.61%	0.37	0.10 0.77	14.46%
**TG 50:1-FA 18:0**	0.72	0.52 0.86	14.22%	0.43	0.15 0.76	20.39%
**TG 50:1-FA 18:1**	0.76	0.58 0.88	10.73%	0.18	0.02 0.74	169.28%
**TG 50:1-FA 20:1**	0.62	0.38 0.81	16.95%			
**TG 50:2-FA 14:0**	0.73	0.53 0.86	5.67%	0.00	0.00 0.00	29.62%
**TG 50:2-FA 14:1**	0.61	0.37 0.80	11.85%			
**TG 50:2-FA 16:0**	0.78	0.62 0.89	4.06%	0.51	0.23 0.78	45.12%
**TG 50:2-FA 16:1**	0.79	0.63 0.89	1.06%	0.62	0.35 0.83	9.83%
**TG 50:2-FA 18:0**	0.72	0.51 0.86	11.06%			
**TG 50:2-FA 18:1**	0.75	0.56 0.87	4.12%	0.52	0.24 0.79	16.11%

**TG 50:2-FA 18:2**	0.80	0.64 0.90	5.56%	0.57	0.30 0.80	45.62%
**TG 50:3-FA 14:0**	0.78	0.61 0.89	3.20%			
**TG 50:3-FA 14:1**	0.64	0.41 0.82	7.86%			
**TG 50:3-FA 16:0**	0.82	0.67 0.91	1.73%	0.26	0.04 0.75	42.58%
**TG 50:3-FA 16:1**	0.78	0.61 0.89	-2.68%	0.56	0.29 0.79	9.54%
**TG 50:3-FA 18:0**	0.78	0.61 0.89	12.09%			
**TG 50:3-FA 18:1**	0.74	0.54 0.87	2.82%	0.43	0.15 0.76	8.76%
**TG 50:3-FA 18:2**	0.79	0.63 0.90	0.64%	0.41	0.14 0.75	16.14%
**TG 50:3-FA 18:3**	0.78	0.60 0.89	10.88%			
**TG 50:3-FA 20:3**	0.66	0.43 0.83	14.95%			
**TG 50:4-FA 14:0**	0.81	0.66 0.91	4.51%			
**TG 50:4-FA 14:1**	0.70	0.48 0.85	3.67%			
**TG 50:4-FA 16:0**	0.80	0.63 0.90	9.28%			
**TG 50:4-FA 16:1**	0.79	0.63 0.89	-4.95%			
**TG 50:4-FA 18:1**	0.74	0.55 0.87	8.11%			
**TG 50:4-FA 18:2**	0.82	0.67 0.91	-0.74%			
**TG 50:4-FA 18:3**	0.77	0.60 0.89	7.47%			
**TG 50:4-FA 20:3**	0.49	0.24 0.74	14.00%			
**TG 50:4-FA 20:4**	0.66	0.44 0.83	27.53%			
**TG 50:5-FA 14:0**	0.79	0.62 0.90	6.16%			
**TG 50:5-FA 16:0**	0.70	0.49 0.85	16.20%			
**TG 50:5-FA 16:1**	0.79	0.63 0.90	-0.22%			
**TG 50:5-FA 18:1**	0.75	0.56 0.88	11.39%			
**TG 50:5-FA 18:2**	0.81	0.65 0.91	5.01%			
**TG 50:5-FA 18:3**	0.78	0.61 0.89	1.32%			
**TG 50:5-FA 20:4**	0.69	0.48 0.85	30.65%			
**TG 50:5-FA 20:5**	0.62	0.38 0.82	22.46%			
**TG 50:6-FA 20:4**	0.76	0.57 0.88	18.36%			
**TG 51:0-FA 16:0**	0.63	0.39 0.81	20.49%			
**TG 51:0-FA 17:0**	0.65	0.43 0.83	21.56%			
**TG 51:0-FA 18:0**	0.57	0.33 0.79	24.50%			
**TG 51:1-FA 15:0**	0.70	0.49 0.85	12.69%			
**TG 51:1-FA 16:0**	0.73	0.53 0.86	14.46%	0.39	0.12 0.74	19.64%
**TG 51:1-FA 17:0**	0.74	0.55 0.87	14.12%	0.59	0.32 0.82	13.23%
**TG 51:1-FA 18:0**	0.68	0.46 0.84	16.27%			
**TG 51:1-FA 18:1**	0.73	0.53 0.86	14.36%	0.43	0.15 0.76	2.46%
**TG 51:2-FA 15:0**	0.74	0.55 0.87	3.23%	0.40	0.15 0.72	2.13%
**TG 51:2-FA 16:0**	0.76	0.58 0.88	5.91%	0.34	0.11 0.68	4.90%
**TG 51:2-FA 16:1**	0.73	0.54 0.86	3.55%			
**TG 51:2-FA 17:0**	0.77	0.60 0.88	6.32%	0.38	0.11 0.76	10.11%
**TG 51:2-FA 18:1**	0.72	0.52 0.86	4.17%	0.64	0.37 0.84	4.86%
**TG 51:2-FA 18:2**	0.77	0.60 0.89	9.13%			
**TG 51:3-FA 15:0**	0.80	0.65 0.90	-0.87%			
**TG 51:3-FA 16:1**	0.76	0.59 0.88	-2.80%			
**TG 51:3-FA 17:0**	0.81	0.65 0.90	1.89%			
**TG 51:3-FA 18:2**	0.80	0.63 0.90	1.58%	0.44	0.17 0.75	10.52%
**TG 51:3-FA 18:3**	0.74	0.54 0.87	12.13%			
**TG 51:4-FA 15:0**	0.81	0.65 0.91	-1.07%			
**TG 51:4-FA 16:1**	0.72	0.52 0.86	-1.70%			
**TG 51:4-FA 18:2**	0.82	0.67 0.91	-2.98%			
**TG 51:4-FA 18:3**	0.78	0.61 0.89	4.96%			

**TG 51:4-FA 20:4**	0.65	0.43 0.82	28.46%			
**TG 51:5-FA 18:2**	0.80	0.63 0.90	-5.43%			
**TG 51:5-FA 18:3**	0.75	0.56 0.88	-2.77%			
**TG 52:0-FA 16:0**	0.70	0.48 0.85	6.25%	0.04	0.00 1.00	25.49%
**TG 52:0-FA 18:0**	0.66	0.43 0.83	1.27%	0.30	0.08 0.66	12.00%
**TG 52:0-FA 20:0**	0.58	0.33 0.79	34.52%			
**TG 52:1-FA 16:0**	0.76	0.57 0.88	1.63%	0.40	0.15 0.71	50.31%
**TG 52:1-FA 16:1**	0.73	0.53 0.86	5.96%			
**TG 52:1-FA 18:0**	0.73	0.54 0.87	2.22%	0.37	0.13 0.70	55.28%
**TG 52:1-FA 18:1**	0.76	0.58 0.88	1.99%	0.35	0.11 0.71	45.83%
**TG 52:1-FA 20:0**	0.69	0.48 0.85	22.05%			
**TG 52:1-FA 20:1**	0.74	0.54 0.87	7.27%			
**TG 52:2-FA 14:0**	0.73	0.53 0.87	3.92%			
**TG 52:2-FA 16:0**	0.77	0.60 0.89	-1.61%	0.42	0.16 0.73	34.08%
**TG 52:2-FA 16:1**	0.71	0.50 0.85	-3.32%	0.48	0.20 0.77	2.94%
**TG 52:2-FA 18:0**	0.78	0.61 0.89	-3.87%	0.74	0.55 0.87	17.92%
**TG 52:2-FA 18:1**	0.76	0.58 0.88	-1.50%	0.33	0.10 0.70	31.30%
**TG 52:2-FA 18:2**	0.81	0.66 0.91	-3.74%	0.63	0.39 0.81	17.99%
**TG 52:2-FA 20:0**	0.73	0.53 0.87	8.95%			
**TG 52:2-FA 20:1**	0.74	0.54 0.87	-0.28%			
**TG 52:2-FA 20:2**	0.71	0.50 0.85	4.22%			
**TG 52:3-FA 14:0**	0.68	0.46 0.84	4.15%			
**TG 52:3-FA 16:0**	0.81	0.66 0.91	-3.86%	0.47	0.21 0.74	25.19%
**TG 52:3-FA 16:1**	0.64	0.42 0.82	-9.15%	0.39	0.13 0.73	15.93%
**TG 52:3-FA 18:0**	0.80	0.63 0.90	0.07%			
**TG 52:3-FA 18:1**	0.78	0.61 0.89	-4.60%	0.51	0.24 0.78	20.45%
**TG 52:3-FA 18:2**	0.82	0.66 0.91	-4.10%	0.46	0.20 0.75	22.33%
**TG 52:3-FA 18:3**	0.79	0.61 0.89	3.19%			
**TG 52:3-FA 20:0**	0.80	0.64 0.90	-2.47%			
**TG 52:3-FA 20:1**	0.75	0.56 0.88	2.66%			
**TG 52:3-FA 20:2**	0.72	0.52 0.86	-0.55%			
**TG 52:3-FA 20:3**	0.72	0.51 0.86	7.09%			
**TG 52:3-FA 22:1**	0.72	0.52 0.86	-6.95%			
**TG 52:4-FA 14:0**	0.65	0.42 0.83	7.68%			
**TG 52:4-FA 16:0**	0.82	0.67 0.91	-3.61%	0.63	0.36 0.84	18.72%
**TG 52:4-FA 16:1**	0.71	0.51 0.86	-11.52%	0.50	0.23 0.77	13.11%
**TG 52:4-FA 18:0**	0.75	0.56 0.88	5.92%			
**TG 52:4-FA 18:1**	0.76	0.58 0.88	-5.49%	0.37	0.11 0.74	13.14%
**TG 52:4-FA 18:2**	0.83	0.68 0.91	-7.28%	0.62	0.36 0.82	20.52%
**TG 52:4-FA 18:3**	0.78	0.61 0.89	1.74%			
**TG 52:4-FA 20:0**	0.83	0.69 0.92	-5.58%			
**TG 52:4-FA 20:2**	0.69	0.48 0.85	-5.72%			
**TG 52:4-FA 20:3**	0.69	0.47 0.84	0.06%			
**TG 52:4-FA 20:4**	0.73	0.53 0.86	13.40%			
**TG 52:4-FA 22:1**	0.68	0.46 0.84	-2.26%			
**TG 52:4-FA 22:4**	0.60	0.37 0.80	19.28%			
**TG 52:5-FA 14:0**	0.69	0.48 0.84	9.43%			
**TG 52:5-FA 16:0**	0.78	0.61 0.89	1.43%			
**TG 52:5-FA 16:1**	0.78	0.60 0.89	-8.85%			
**TG 52:5-FA 18:1**	0.72	0.52 0.86	-2.38%			
**TG 52:5-FA 18:2**	0.81	0.66 0.91	-7.36%	0.02	0.00 1.00	16.67%

**TG 52:5-FA 18:3**	0.78	0.61 0.89	-1.83%			
**TG 52:5-FA 20:3**	0.64	0.40 0.82	-3.69%			
**TG 52:5-FA 20:4**	0.73	0.54 0.86	14.60%			
**TG 52:5-FA 20:5**	0.78	0.61 0.89	12.84%			
**TG 52:5-FA 22:5**	0.79	0.63 0.90	8.14%			
**TG 52:6-FA 16:0**	0.76	0.58 0.88	7.97%			
**TG 52:6-FA 16:1**	0.77	0.59 0.88	-5.11%			
**TG 52:6-FA 18:1**	0.69	0.47 0.85	8.75%			
**TG 52:6-FA 18:2**	0.78	0.60 0.89	-1.39%			
**TG 52:6-FA 18:3**	0.75	0.56 0.88	-1.77%			
**TG 52:6-FA 20:4**	0.68	0.46 0.84	7.69%			
**TG 52:6-FA 20:5**	0.82	0.68 0.91	8.29%			
**TG 52:6-FA 22:6**	0.82	0.67 0.91	4.83%			
**TG 52:7-FA 16:0**	0.65	0.42 0.82	21.56%			
**TG 52:7-FA 18:1**	0.69	0.48 0.85	11.87%			
**TG 52:7-FA 22:6**	0.81	0.66 0.91	-0.20%			
**TG 52:8-FA 16:1**	0.77	0.60 0.88	14.28%			
**TG 52:8-FA 18:2**	0.75	0.56 0.88	9.81%			
**TG 53:0-FA 16:0**	0.83	0.68 0.91	2.76%			
**TG 53:1-FA 16:0**	0.70	0.49 0.85	10.27%			
**TG 53:1-FA 17:0**	0.69	0.48 0.85	6.53%			
**TG 53:1-FA 18:0**	0.68	0.46 0.84	7.49%			
**TG 53:1-FA 18:1**	0.72	0.51 0.86	10.39%			
**TG 53:2-FA 16:0**	0.69	0.47 0.84	4.72%			
**TG 53:2-FA 17:0**	0.74	0.54 0.87	-2.01%			
**TG 53:2-FA 18:1**	0.73	0.53 0.86	1.45%	0.31	0.08 0.69	0.50%
**TG 53:2-FA 18:2**	0.76	0.57 0.88	-0.18%			
**TG 53:3-FA 16:0**	0.71	0.50 0.86	-0.31%			
**TG 53:3-FA 17:0**	0.80	0.63 0.90	-3.46%			
**TG 53:3-FA 18:2**	0.79	0.62 0.90	-2.85%			
**TG 53:4-FA 16:0**	0.71	0.50 0.86	7.92%			
**TG 53:4-FA 17:0**	0.82	0.67 0.91	-3.37%			
**TG 53:4-FA 18:2**	0.81	0.65 0.91	-7.37%	0.38	0.14 0.69	0.10%
**TG 53:4-FA 18:3**	0.73	0.53 0.87	1.01%			
**TG 53:4-FA 20:4**	0.75	0.56 0.87	12.84%			
**TG 53:5-FA 20:4**	0.72	0.52 0.86	7.49%			
**TG 53:6-FA 20:4**	0.74	0.55 0.87	1.39%			
**TG 54:0-FA 16:0**	0.65	0.41 0.83	20.57%			
**TG 54:0-FA 18:0**	0.57	0.32 0.79	-11.08%	0.50	0.25 0.76	7.90%
**TG 54:1-FA 16:0**	0.70	0.49 0.85	11.98%			
**TG 54:1-FA 18:0**	0.59	0.34 0.79	-15.92%	0.53	0.28 0.77	16.02%
**TG 54:1-FA 18:1**	0.67	0.44 0.83	-6.37%	0.21	0.03 0.68	4.73%
**TG 54:1-FA 20:0**	0.67	0.44 0.84	14.45%			
**TG 54:1-FA 20:1**	0.74	0.54 0.87	1.17%			
**TG 54:2-FA 16:0**	0.75	0.55 0.88	-1.72%	0.00	0.00 0.00	24.82%
**TG 54:2-FA 18:0**	0.69	0.47 0.85	-9.22%	0.61	0.38 0.81	13.39%
**TG 54:2-FA 18:1**	0.69	0.48 0.85	-6.12%	0.38	0.14 0.71	16.87%
**TG 54:2-FA 18:2**	0.75	0.55 0.87	-12.08%	0.35	0.11 0.70	24.69%
**TG 54:2-FA 20:0**	0.73	0.53 0.87	-0.39%			
**TG 54:2-FA 20:1**	0.76	0.57 0.88	-0.38%	0.08	0.00 0.94	15.20%
**TG 54:2-FA 20:2**	0.71	0.50 0.86	-0.51%			

**TG 54:3-FA 16:0**	0.74	0.54	0.87	-3.23%	0.20	0.02	0.71	22.23%
**TG 54:3-FA 16:1**	0.65	0.42	0.83	-10.74%				
**TG 54:3-FA 18:0**	0.77	0.58	0.89	-10.92%	0.59	0.36	0.79	13.44%
**TG 54:3-FA 18:1**	0.65	0.42	0.83	-5.29%	0.35	0.11	0.70	14.20%
**TG 54:3-FA 18:2**	0.76	0.57	0.88	-10.87%	0.34	0.11	0.69	14.63%
**TG 54:3-FA 18:3**	0.74	0.54	0.87	-2.97%				
**TG 54:3-FA 20:1**	0.77	0.59	0.89	-3.77%				
**TG 54:3-FA 20:2**	0.68	0.46	0.84	-4.96%				
**TG 54:3-FA 20:3**	0.68	0.46	0.84	-1.99%				
**TG 54:4-FA 16:0**	0.72	0.51	0.86	-1.99%	0.32	0.09	0.69	8.35%
**TG 54:4-FA 16:1**	0.62	0.37	0.81	-8.91%				
**TG 54:4-FA 18:0**	0.79	0.62	0.90	-11.96%	0.33	0.09	0.71	33.19%
**TG 54:4-FA 18:1**	0.71	0.50	0.86	-5.83%	0.49	0.23	0.75	11.84%
**TG 54:4-FA 18:2**	0.74	0.54	0.87	-7.89%	0.51	0.25	0.76	11.39%
**TG 54:4-FA 18:3**	0.74	0.53	0.87	-3.65%				
**TG 54:4-FA 20:1**	0.74	0.55	0.87	-3.56%				
**TG 54:4-FA 20:2**	0.66	0.43	0.84	-7.04%				
**TG 54:4-FA 20:3**	0.67	0.45	0.84	-4.75%	0.33	0.10	0.70	7.32%
**TG 54:4-FA 20:4**	0.75	0.57	0.88	0.90%	0.38	0.14	0.70	15.38%
**TG 54:4-FA 22:1**	0.75	0.57	0.88	-4.82%				
**TG 54:4-FA 22:4**	0.73	0.53	0.86	7.57%				
**TG 54:5-FA 16:0**	0.75	0.57	0.88	1.34%	0.16	0.01	0.74	-8.05%
**TG 54:5-FA 16:1**	0.66	0.44	0.83	-3.84%				
**TG 54:5-FA 18:0**	0.74	0.54	0.87	-4.27%				
**TG 54:5-FA 18:1**	0.72	0.51	0.86	-4.29%	0.49	0.24	0.74	11.40%
**TG 54:5-FA 18:2**	0.76	0.57	0.88	-7.37%	0.50	0.25	0.75	9.37%
**TG 54:5-FA 18:3**	0.71	0.50	0.86	-3.26%				
**TG 54:5-FA 20:2**	0.68	0.45	0.85	-7.50%				
**TG 54:5-FA 20:3**	0.66	0.43	0.84	-6.40%				
**TG 54:5-FA 20:4**	0.75	0.56	0.87	2.14%	0.36	0.11	0.71	18.47%
**TG 54:5-FA 20:5**	0.82	0.67	0.91	6.28%				
**TG 54:5-FA 22:1**	0.69	0.47	0.85	-1.70%				
**TG 54:5-FA 22:4**	0.69	0.49	0.84	2.05%				
**TG 54:5-FA 22:5**	0.81	0.66	0.90	1.51%				
**TG 54:6-FA 16:0**	0.80	0.65	0.90	0.60%				
**TG 54:6-FA 16:1**	0.77	0.59	0.88	-2.90%				
**TG 54:6-FA 18:1**	0.70	0.48	0.85	-0.63%				
**TG 54:6-FA 18:2**	0.74	0.54	0.87	-4.86%	0.40	0.16	0.71	13.77%
**TG 54:6-FA 18:3**	0.74	0.54	0.87	-2.41%				
**TG 54:6-FA 20:3**	0.65	0.41	0.83	-7.91%				
**TG 54:6-FA 20:4**	0.72	0.52	0.86	0.06%				
**TG 54:6-FA 20:5**	0.83	0.69	0.92	3.35%				
**TG 54:6-FA 22:5**	0.79	0.63	0.89	-4.87%				
**TG 54:6-FA 22:6**	0.89	0.79	0.94	1.13%				
**TG 54:7-FA 16:1**	0.85	0.73	0.93	-4.81%				
**TG 54:7-FA 18:1**	0.68	0.46	0.84	4.56%				
**TG 54:7-FA 18:2**	0.71	0.50	0.86	4.00%				
**TG 54:7-FA 18:3**	0.70	0.48	0.85	5.30%				
**TG 54:7-FA 20:4**	0.68	0.46	0.84	-0.02%				
**TG 54:7-FA 20:5**	0.81	0.65	0.90	1.33%				
**TG 54:7-FA 22:5**	0.76	0.58	0.88	-5.53%				

**TG 54:7-FA 22:6**	0.90	0.80	0.95	-6.26%				
**TG 54:8-FA 18:2**	0.71	0.50	0.86	4.61%				
**TG 54:8-FA 18:3**	0.65	0.42	0.83	9.65%				
**TG 54:8-FA 20:4**	0.64	0.40	0.82	4.00%				
**TG 54:8-FA 20:5**	0.80	0.64	0.90	-0.17%				
**TG 54:8-FA 22:6**	0.86	0.74	0.93	-6.76%				
**TG 55:1-FA 16:0**	0.82	0.68	0.91	-5.87%				
**TG 55:1-FA 18:1**	0.72	0.51	0.86	-1.70%				
**TG 55:2-FA 18:1**	0.65	0.41	0.83	9.27%				
**TG 55:2-FA 18:2**	0.73	0.53	0.87	-1.94%				
**TG 55:3-FA 18:1**	0.64	0.40	0.83	4.91%				
**TG 55:3-FA 18:2**	0.74	0.54	0.87	-0.39%				
**TG 55:4-FA 18:1**	0.63	0.39	0.82	0.49%	0.17	0.01	0.75	0.36%
**TG 55:4-FA 18:2**	0.71	0.49	0.86	-2.66%				
**TG 55:5-FA 18:1**	0.63	0.38	0.82	1.59%	0.14	0.01	0.75	5.03%
**TG 55:5-FA 18:2**	0.66	0.43	0.84	-5.07%				
**TG 55:5-FA 20:4**	0.70	0.49	0.85	-2.16%	0.55	0.31	0.76	21.47%
**TG 55:7-FA 15:0**	0.72	0.51	0.86	-3.90%	0.23	0.04	0.69	50.53%
**TG 55:7-FA 22:6**	0.86	0.73	0.93	-4.79%				
**TG 56:10-FA 18:2**	0.72	0.51	0.86	4.43%				
**TG 56:1-FA 16:0**	0.62	0.37	0.82	7.24%				
**TG 56:1-FA 18:1**	0.65	0.41	0.84	2.37%	0.28	0.05	0.74	1.79%
**TG 56:2-FA 16:0**	0.67	0.44	0.84	0.99%				
**TG 56:2-FA 18:0**	0.73	0.52	0.87	-7.58%				
**TG 56:2-FA 20:0**	0.60	0.35	0.81	-1.98%				
**TG 56:2-FA 20:1**	0.68	0.45	0.85	-4.42%				
**TG 56:3-FA 16:0**	0.66	0.43	0.83	3.71%				
**TG 56:3-FA 18:0**	0.75	0.55	0.88	-9.56%	0.09	0.00	0.89	40.20%
**TG 56:3-FA 18:1**	0.65	0.40	0.83	-7.66%	0.25	0.05	0.68	9.69%
**TG 56:3-FA 18:2**	0.69	0.46	0.85	-4.90%				
**TG 56:3-FA 20:0**	0.64	0.39	0.83	-2.94%				
**TG 56:3-FA 20:1**	0.67	0.43	0.84	-5.49%	0.20	0.02	0.72	9.15%
**TG 56:3-FA 20:2**	0.67	0.43	0.84	-9.03%				
**TG 56:4-FA 16:0**	0.66	0.44	0.83	-2.85%				
**TG 56:4-FA 18:0**	0.74	0.54	0.87	-10.22%	0.23	0.04	0.69	30.49%
**TG 56:4-FA 18:1**	0.68	0.45	0.84	-8.52%	0.07	0.00	0.96	15.13%
**TG 56:4-FA 18:2**	0.67	0.44	0.84	-7.42%				
**TG 56:4-FA 20:1**	0.66	0.43	0.84	-5.18%				
**TG 56:4-FA 20:2**	0.66	0.43	0.84	-12.01%				
**TG 56:4-FA 20:3**	0.68	0.45	0.84	-11.99%				
**TG 56:4-FA 20:4**	0.77	0.59	0.89	-13.15%	0.34	0.12	0.67	28.38%
**TG 56:4-FA 22:4**	0.74	0.55	0.87	-5.40%				
**TG 56:5-FA 16:0**	0.73	0.53	0.87	-3.05%				
**TG 56:5-FA 18:0**	0.74	0.55	0.88	-13.91%	0.39	0.16	0.69	-7.98%
**TG 56:5-FA 18:1**	0.69	0.47	0.85	-10.35%	0.14	0.01	0.79	13.95%
**TG 56:5-FA 18:2**	0.76	0.57	0.88	-9.66%				
**TG 56:5-FA 20:1**	0.63	0.39	0.82	-2.82%				
**TG 56:5-FA 20:2**	0.73	0.53	0.87	-11.96%				
**TG 56:5-FA 20:3**	0.59	0.34	0.80	-12.06%				
**TG 56:5-FA 20:4**	0.71	0.50	0.86	-15.95%	0.36	0.12	0.70	6.23%
**TG 56:5-FA 22:4**	0.73	0.54	0.87	-5.51%				

**TG 56:5-FA 22:5**	0.83	0.68	0.91	-4.65%				
**TG 56:6-FA 16:0**	0.81	0.65	0.90	-6.72%				
**TG 56:6-FA 18:0**	0.76	0.58	0.88	-11.50%				
**TG 56:6-FA 18:1**	0.73	0.53	0.87	-7.13%	0.13	0.00	0.82	5.27%
**TG 56:6-FA 18:2**	0.75	0.55	0.88	-12.90%				
**TG 56:6-FA 18:3**	0.67	0.44	0.84	-4.12%				
**TG 56:6-FA 20:2**	0.65	0.41	0.83	-5.16%				
**TG 56:6-FA 20:3**	0.68	0.46	0.85	-12.05%				
**TG 56:6-FA 20:4**	0.68	0.46	0.85	-10.26%	0.25	0.05	0.68	1.06%
**TG 56:6-FA 20:5**	0.78	0.60	0.89	-3.49%				
**TG 56:6-FA 22:4**	0.71	0.50	0.86	-7.69%				
**TG 56:6-FA 22:5**	0.81	0.65	0.90	-6.87%				
**TG 56:6-FA 22:6**	0.87	0.76	0.94	-5.02%				
**TG 56:7-FA 16:0**	0.85	0.71	0.92	-8.05%				
**TG 56:7-FA 16:1**	0.61	0.37	0.81	-11.44%				
**TG 56:7-FA 18:0**	0.76	0.57	0.88	-7.76%				
**TG 56:7-FA 18:1**	0.79	0.62	0.90	-6.73%				
**TG 56:7-FA 18:2**	0.79	0.61	0.90	-8.01%				
**TG 56:7-FA 18:3**	0.66	0.43	0.84	-6.16%				
**TG 56:7-FA 20:3**	0.64	0.40	0.83	-8.81%	0.00	0.00	0.00	-11.55%
**TG 56:7-FA 20:4**	0.66	0.43	0.84	-9.92%	0.40	0.16	0.71	1.55%
**TG 56:7-FA 20:5**	0.78	0.60	0.89	-0.70%				
**TG 56:7-FA 22:4**	0.69	0.48	0.85	-3.16%				
**TG 56:7-FA 22:5**	0.81	0.66	0.91	-8.32%				
**TG 56:7-FA 22:6**	0.88	0.78	0.94	-8.36%	0.15	0.01	0.79	7.82%
**TG 56:8-FA 16:0**	0.82	0.68	0.91	-5.15%				
**TG 56:8-FA 16:1**	0.73	0.53	0.86	-15.68%				
**TG 56:8-FA 18:1**	0.74	0.54	0.87	-2.78%				
**TG 56:8-FA 18:2**	0.77	0.60	0.89	-6.72%				
**TG 56:8-FA 18:3**	0.69	0.46	0.85	-4.53%				
**TG 56:8-FA 20:4**	0.61	0.37	0.81	-7.32%	0.47	0.22	0.74	1.57%
**TG 56:8-FA 20:5**	0.73	0.53	0.87	-0.33%				
**TG 56:8-FA 22:5**	0.77	0.59	0.89	-9.41%				
**TG 56:8-FA 22:6**	0.86	0.74	0.93	-9.85%				
**TG 56:9-FA 18:3**	0.76	0.57	0.88	-4.36%				
**TG 56:9-FA 20:4**	0.64	0.41	0.83	1.20%				
**TG 56:9-FA 20:5**	0.69	0.47	0.85	-0.49%				
**TG 56:9-FA 22:6**	0.80	0.65	0.90	-11.16%				
**TG 57:2-FA 18:1**	0.70	0.49	0.85	-7.20%				
**TG 57:3-FA 18:2**	0.65	0.42	0.83	-7.87%				
**TG 58:10-FA 18:2**	0.67	0.45	0.84	-6.90%				
**TG 58:10-FA 20:4**	0.71	0.51	0.86	-5.27%				
**TG 58:10-FA 20:5**	0.77	0.60	0.89	-3.63%				
**TG 58:10-FA 22:5**	0.63	0.41	0.81	-5.75%				
**TG 58:10-FA 22:6**	0.75	0.57	0.88	-8.33%				
**TG 58:2-FA 18:1**	0.61	0.35	0.82	-11.70%				
**TG 58:3-FA 18:1**	0.52	0.24	0.78	-0.47%				
**TG 58:5-FA 18:1**	0.68	0.46	0.85	-10.17%				
**TG 58:6-FA 16:0**	0.65	0.41	0.83	2.40%				
**TG 58:6-FA 18:0**	0.72	0.51	0.86	-12.20%				
**TG 58:6-FA 18:1**	0.68	0.45	0.85	-9.30%				

**TG 58:6-FA 20:4**	0.62	0.37	0.82	-13.14%
**TG 58:6-FA 22:4**	0.64	0.40	0.83	-14.69%
**TG 58:6-FA 22:5**	0.73	0.53	0.87	-10.48%
**TG 58:7-FA 16:0**	0.46	0.21	0.74	-1.66%
**TG 58:7-FA 18:0**	0.75	0.56	0.88	-12.94%
**TG 58:7-FA 18:1**	0.71	0.49	0.86	-8.79%
**TG 58:7-FA 18:2**	0.73	0.53	0.87	-9.69%
**TG 58:7-FA 20:4**	0.60	0.35	0.80	-12.17%
**TG 58:7-FA 22:4**	0.61	0.37	0.81	-15.65%
**TG 58:7-FA 22:5**	0.69	0.48	0.85	-11.73%
**TG 58:7-FA 22:6**	0.83	0.68	0.91	-10.99%
**TG 58:8-FA 18:1**	0.73	0.52	0.87	-11.45%
**TG 58:8-FA 18:2**	0.78	0.61	0.89	-12.06%
**TG 58:8-FA 20:3**	0.71	0.51	0.86	-9.22%
**TG 58:8-FA 20:4**	0.66	0.43	0.83	-10.32%
**TG 58:8-FA 22:5**	0.75	0.55	0.87	-9.94%
**TG 58:8-FA 22:6**	0.81	0.66	0.91	-10.60%
**TG 58:9-FA 18:1**	0.70	0.48	0.85	-9.56%
**TG 58:9-FA 18:2**	0.71	0.50	0.86	-10.35%
**TG 58:9-FA 20:4**	0.71	0.51	0.86	-4.16%
**TG 58:9-FA 22:5**	0.66	0.44	0.83	-5.53%
**TG 58:9-FA 22:6**	0.79	0.63	0.90	-9.56%
**TG 60:10-FA 22:5**	0.54	0.30	0.77	-11.72%
**TG 60:10-FA 22:6**	0.80	0.64	0.90	-12.33%
**TG 60:11-FA 22:5**	0.61	0.37	0.80	-8.16%
**TG 60:11-FA 22:6**	0.78	0.61	0.89	-8.36%
**TG 60:12-FA 22:6**	0.74	0.56	0.87	-13.76%

Δ Numbers represent the mean change in lipid concentration over the 6 week period, expressed as percentage change from the baseline concentration.

Table S3. Reproducibility of lipids in plasma and erythrocytes, stratified by lipid class

	All lipids		Lipids measured in both sample types	
	Plasma	Erythrocytes	Plasma	Erythrocytes
Cholesteryl esters	0.64	0.35	0.72	0.35
Ceramides	0.56	0.81	0.64	0.81
Diacylglycerols	0.34	0.49	0.48	0.62
Dihydroceramides	0.31	0.71	-	-
Free fatty acids	0.65	0.55	0.65	0.52
Hexosylceramides	0.58	0.71	0.61	0.71
Lactoceramides	0.66	0.59	0.78	0.44
Lysophosphatidylcholines	0.56	0.69	0.57	0.69
Lysophosphatidylethanolamines	0.48	0.73	0.46	0.87
Phosphatidylcholines	0.66	0.71	0.66	0.72
Phosphatidylethanolamines	0.50	0.63	0.53	0.68
Sphingomyelins	0.76	0.88	0.77	0.88
Triacylglycerols	0.71	0.40	0.72	0.40

Numbers represent the median intra-class correlation coefficients (ICC) of the ICCs of the individual lipids within a class. N=230 lipids were measured in both plasma and erythrocytes.
